# Catheter ablation of premature ventricular contractions originating from kissing papillary muscles

**DOI:** 10.1002/ccr3.4955

**Published:** 2021-10-18

**Authors:** Takumi Yamada, Krittapoom Akrawinthawong

**Affiliations:** ^1^ Cardiovascular Division University of Minnesota Minneapolis Minnesota USA; ^2^ Prairie Heart Institute Carbondale Illinois USA

**Keywords:** catheter ablation, papillary muscle, premature ventricular contraction

## Abstract

Very thick left ventricular papillary muscles (PAMs) may kiss each other and premature ventricular contractions (PVCs) can originate from the sides of the PAMs facing each other. In such a setting, mapping of those PVCs is confusing and rendering catheter ablation challenging.

## Graphical abstract

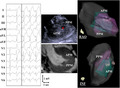

A 73‐year‐old woman with premature ventricular contractions (PVCs) had very thick left ventricular papillary muscles (PAMs) kissing each other. The PVC origin at the septal side of the anterolateral PAM that faced the posteromedial PAM, rendered mapping confusing. This case illustrated an unusual challenge in catheter ablation of PAM PVCs.

A 73‐year‐old woman with symptomatic idiopathic premature ventricular contractions (PVCs) exhibiting a right bundle branch block and right inferior axis QRS morphology (Figure 1) underwent electrophysiological testing. Pre‐procedural cardiac magnetic resonance imaging and intracardiac echocardiography revealed very thick posteromedial and anterolateral papillary muscles (PPM and APM, respectively) in the left ventricle that were kissing each other during systole (Figure 1). Activation mapping during the PVCs was confusing because the earliest ventricular activation was recorded in the middle between the PPM and APM. Pace mapping was helpful for recognizing the mapping catheter's location on the PPM or APM because the QRS axis dramatically changed between them (Figure 1). Radiofrequency catheter ablation was successful on the septal side of the APM where a highly matched pace map was obtained.

**FIGURE 1:**
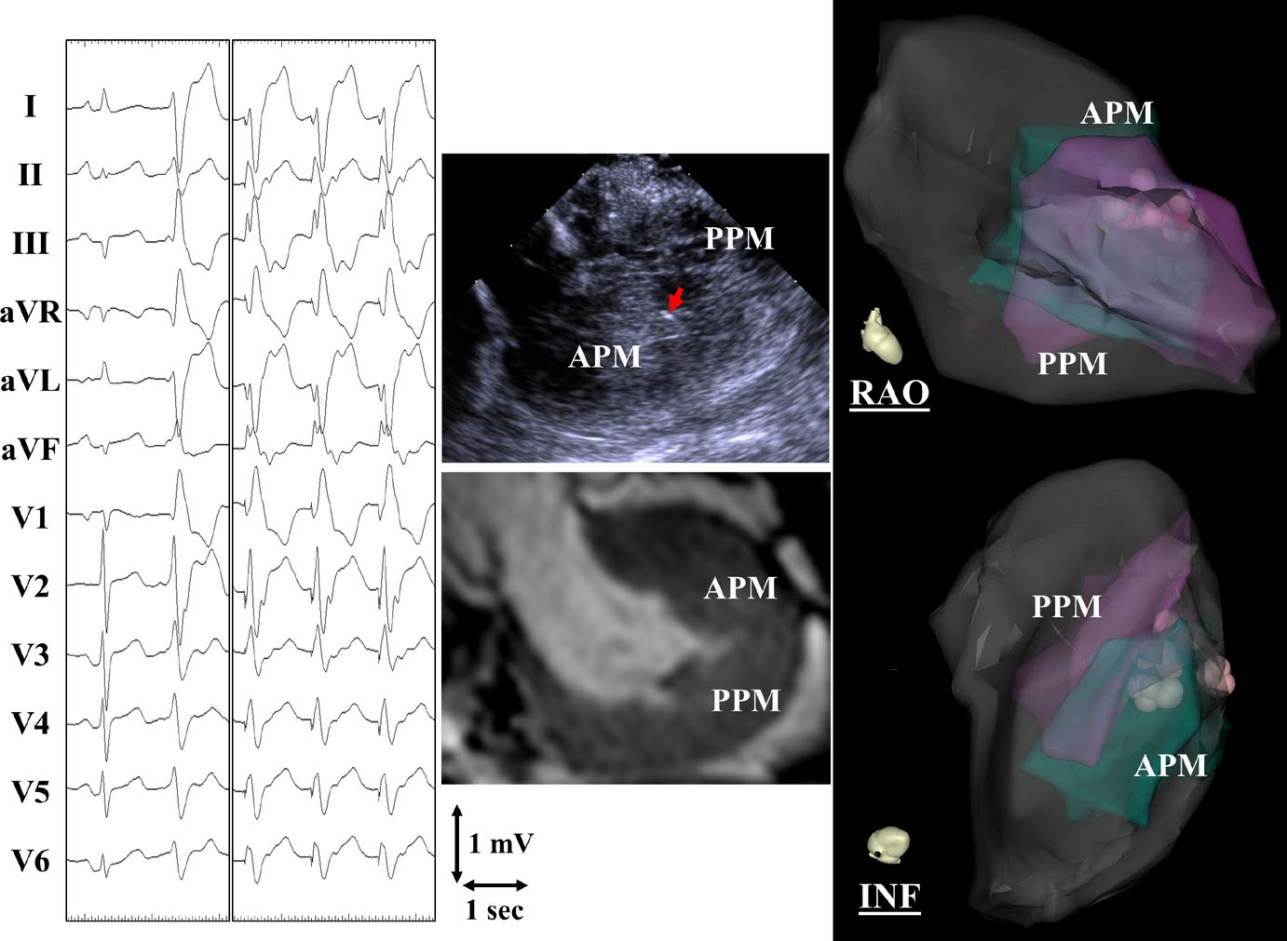
Twelve‐lead electrocardiograms exhibiting a premature ventricular contraction and pace map (left panels), two‐dimensional images of the intracardiac echocardiography (ICE) and cardiac magnetic resonance imaging exhibiting thick papillary muscles in the left ventricle (middle upper and lower panels, respectively), and three‐dimensional maps merged with ICE images exhibiting the successful ablation site (right panels). APM, anterolateral papillary muscle; INF, inferior; PPM, posteromedial papillary muscle; RAO, right anterior oblique view

Catheter ablation of ventricular arrhythmias originating from the papillary muscles (PAMs) is often challenging because their origins are located deep inside thick PAMs.^1^, ^2^ However, in this case, mapping the PAM PVCs was challenging because a very thick PPM and APM were kissing each other, and the PVC origin was located on the septal side of the APM facing the PPM. This case illustrated an unusual challenge and emphasized the importance of cardiac imaging in catheter ablation of PAM PVCs.

## CONFLICT OF INTEREST

The authors declare no conflict of interest.

## AUTHOR CONTRIBUTIONS

Both authors made a substantial contribution to the preparation of this manuscript and approved the final version for submission. TY: drafted the manuscript. KA: acquired the images and performed the literature search.

## ETHICAL APPROVAL

Written informed consent was obtained from the patient. This case study was completed at the University of Alabama at Birmingham. The Institutional Review Board approved all types of the studies regarding ventricular arrhythmias originating from the papillary muscles.

## CONSENT

The authors have confirmed during the submission that the patient consent has been signed and collected in accordance with the journal's patient consent policy.

## Data Availability

The data that support the findings of this study are available from the corresponding author upon reasonable request.
